# Time-resolved mr venography pre catheter-based ablation for atrial fibrillation

**DOI:** 10.1186/1532-429X-13-S1-P234

**Published:** 2011-02-02

**Authors:** Michael Schonberger, Aya Kino, Andrada Popescu, Maurizio Galizia, Jeremy Collins, Timothy Carroll, James Carr

**Affiliations:** 1 Northwestern University, Chicago, IL, USA

## Objective

The purpose of this study was to evaluate Time-resolved MR venography (TR-MRV) of the pulmonary venous circulation using the time-resolved angiography with interleaved stochastic trajectories (TWIST) method of time-resolved MRA (TR-MRA) and compare it with the more commonly used conventional Contrast enhanced magnetic resonance angiography (CE-MRA) approach in atrial fibrillation patients referred for pre-ablation pulmonary vein mapping.

## Background

Catheter-based ablation of the pulmonary veins prevents recurrence of atrial fibrillation in 70-80% of patients during the first year of follow-up^1,2^. CE-MRA depicts the left atrium and pulmonary veins with high spatial resolution, enabling accurate measurement of pulmonary vein ostia to be made with depiction of their relationship to other structures.^1,3^ Conventional CE-MRA however requires timing of contrast enhancement and produces images with overlap of venous and arterial structures, potentially obscuring pulmonary vein ostia. TR-MRA is an alternative to conventional CE-MRA and has been used successfully in other vascular territories.^4^ Such an approach may be particularly advantageous in the pulmonary circulation with its rapid arteriovenous transit time, allowing acquisition of pure pulmonary venous phase images with a simpler imaging protocol.

## Material and methods

26 patients (15 males; age 60.0 ± 12.7y) referred for pre-ablation pulmonary vein mapping underwent both conventional CE-MRA and TR-MRV with TWIST. Imaging was performed on a 1.5 Tesla (MAGNETOM Avanto, Siemens Healthcare) MRI scanner. Source partition and MIP images were evaluated. ***Quantitative Analysis:*** Pulmonary vein ostium orthogonal dimensions were measured using double oblique multiplanar reformatting. ***Qualitative Analysis:*** For qualitative analysis, both source partition images and MIP images were assessed by two observers. Pulmonary vein conspicuity was scored on a scale of 1-4 (1=poor, 2=fair, 3=good, 4=excellent). The number of pulmonary veins (3 veins= common ostium, 4 veins = normal, 5 veins=accessory vein) was recorded.

## Results

**Figure 1:**
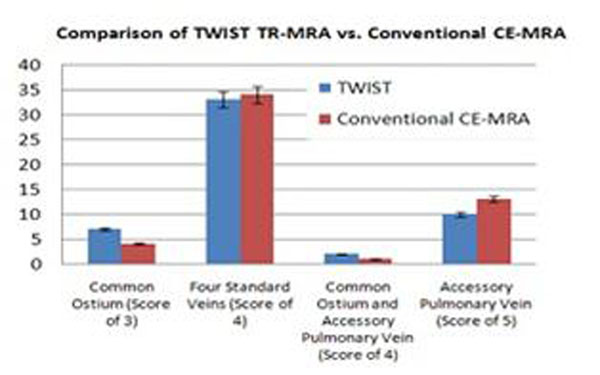
Comparison of accessory vein and common ostium detecting ability using a bar graph to quantify differences between conventional CE-MRA and TWIST TR-MRA. However, TWIST was superior in detecting common ostiums and slightly better than the conventional CE-MRA technique at identifying patients with both common ostiums and accessory veins. Finally, CE-MRA was slightly better overall for visualizing the pulmonary veins but had a greater margin of error. The TWIST technique, on the other hand, had slightly worse overall results but was more consistent in yielding high quality visualizations of the veins. The CE-MRA was able to identify the focal ostial stenosis present in one patient during the qualitative analysis while the TWISE was not.

Orthogonal venous diameters were comparable for both TR-MRV and conventional CEMRA (1.34cm *±* 0.37 vs 1.38cm ± 0.36, respectively); see Table 1. Visualization of pulmonary vein anatomy and variant anatomy was also similar for both techniques (fig 1).

**Table 1:** Comparison of the mean pulmonary diameter ± standard deviation (maximum; minimum values for TWIST and Conventional CE-MRA respectively.

	TWIST (Time-Resolved MRA)	Conventional CE-MRA	P-values
RUPV			
cc	1.58 ± 0.27 (2.08;1.2)	1.68 ± 0.24 (2.08;1.13)	0.069
ap	1.13 ± 0.23 (1.52;0.63)	1.16 ± 0.19 (1.66;0.88)	0.521
RLPV			
cc	1.69 ± 0.19 (2.06;1.41)	1.72 ± 0.16 (2.14;1.49)	0.307
ap	1.22 ± 0.23 (1.65;0.79)	1.32 ± 0.24 (1.84;0.98)	0.005
LUPV			
cc	1.51 ± 0.33 (2.14;0.47)	1.53 ± 0.31 (2.26;0.59)	0.456
ap	1.05 ± 0.28 (1.73;0.35)	1.09 ± 0.25 (1.69;0.53)	0.168
LLPV			
cc	1.54 ± 0.33 (2.64;1.04)	1.57 ± 0.28 (2.34;1.1)	0.573
ap	1.00 ± 0.27 (1.63;0.52)	0.97 ± 0.23 (1.47;0.49)	0.286

All values in cm. cc = measures in cranial-caudal direction; ap = measures in anterior-posterior direction. The t-test revealed a value of 0.004933 which is much less that the level for statistical significance of 0.05. Therefore, the difference between the measuring ability of TWISE and Conventional CE-MRA is not statistically significant.

## Conclusion

We have demonstrated that TR-MRV using TWIST produces comparable anatomic images and pulmonary venous dimensions to the more widely used CEMRA technique. TR-MRV improves arterio-venous separation producing high resolution pulmonary venous phase images without arterial overlap.
